# Supplementary material for: Single-cell and chromatin accessibility profiling reveals regulatory programs of pathogenic Th2 cells in allergic asthma
Source: Nat Commun. 2025 Mar 15;16:2565. doi: 10.1038/s41467-025-57590-3 (PMC11910648; doi:10.1038/s41467-025-57590-3)
Supplement: Supplementary file 1 — Supplementary Information [file 41467_2025_57590_MOESM1_ESM.pdf]

Supplementary figures

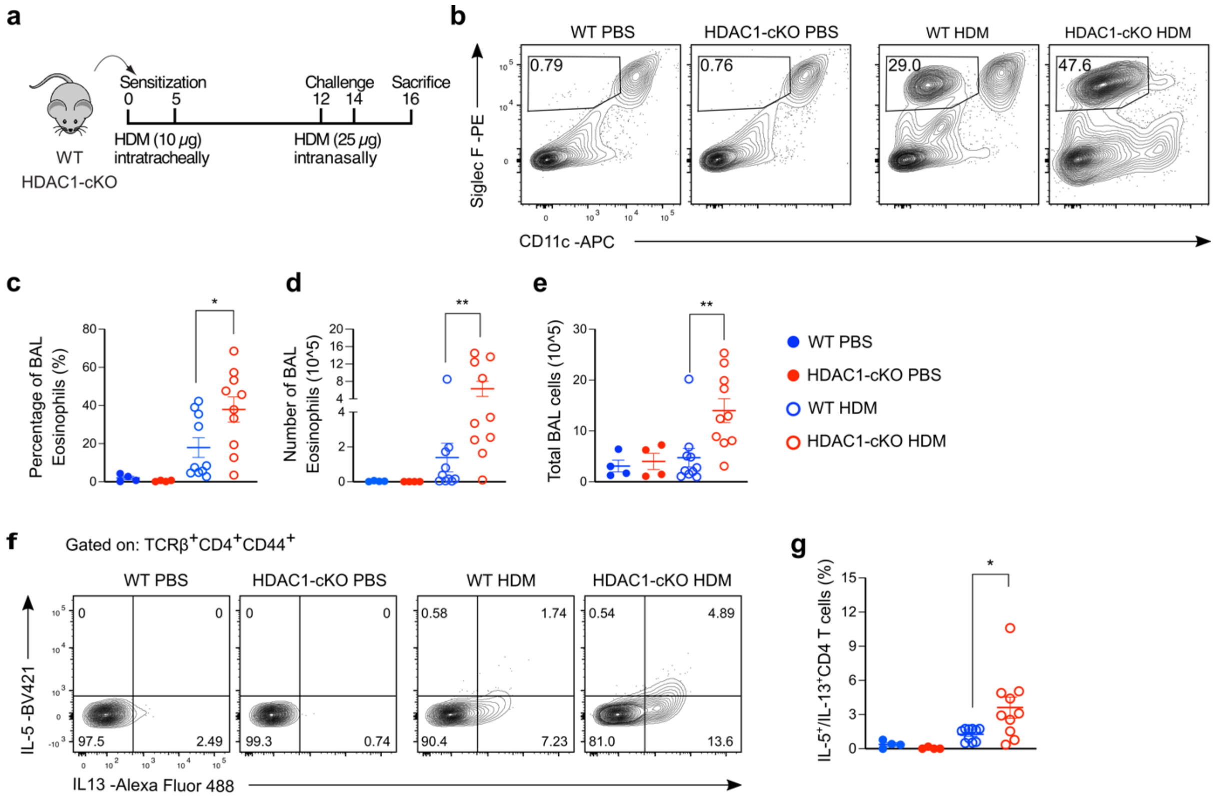

Supplementary Fig.1| HDAC1 is essential to restrict HDM-induced airway inflammation. a, Model of HDM-induced allergic airway inflammation. WT (HDAC1$^{f/f}$ x CD4-Cre$^{-/-}$) and HDAC1-cKO (HDAC1$^{f/f}$ x CD4-Cre$^{+/-}$) mice were sensitised with 10 µg HDM in 40 µl PBS on days 0 and 5. The mice were challenged on days 12 and 14 with 25 µg HDM in 40 µl PBS and euthanised on day 16. Control mice received 40 µl PBS alone during the sensitisation and challenge periods. **b,** Representative flow cytometry plots showing eosinophils (Siglec F^{+}CD11c^{-}) in BAL of the mice with the indicated genotype. **c,** Graph shows the frequency of eosinophils in **b**. **d,e,** Graphs showing the summary of total number of BAL eosinophils (**d**) and the total number of cells in BAL (**e**). **f,g,** *Ex vivo* restimulation of lung cells from WT and HDAC1-cKO control and diseased mice. Lung cells were restimulated with PMA and ionomycin in the presence of GolgiStop and GolgiPlug for 4 hours followed by cytokine analyses by flow cytometry. **f,** Representative flow cytometry plots showing IL-5 and IL-13 expression in lung Th cells (gated on TCRβ^{+}CD4^{+}CD44^{+}). **g,** Graph shows the frequency of IL-5 and IL-13 co-expressing cells in **f.** Data are pooled from two independent experiments (PBS groups: n = 4; HDM groups: n = 10) with each symbol representing one mouse. Data are presented as the mean ± SEM and statistical analysis was performed using a two-tailed Mann-Whitney U test. $^{*}P$<0.05, $^{**}P$<0.01. WT, wild type; HDAC1-cKO, HDAC1-conditional knockout; PBS, phosphate-buffered saline; HDM, house dust mite; BAL, bronchoalveolar lavage; PMA, phorbol 12-myristate 13-acetate; Th, T helper.
Source data (**c,d,e,g**) are provided as a Source Data file.

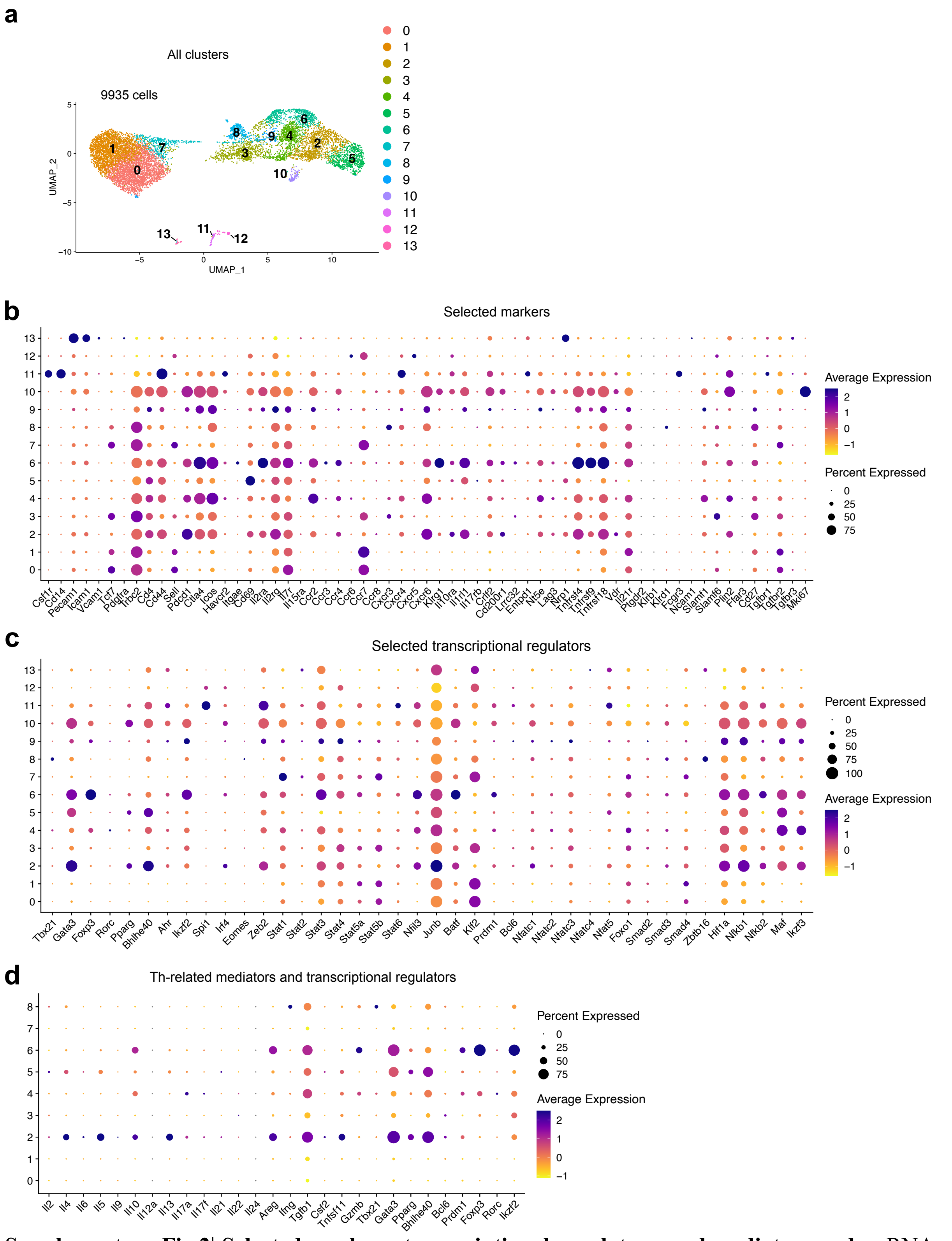

Supplementary Fig.2| Selected markers, transcriptional regulators, and mediators. a-d, scRNA-seq analysis of lung CD4$^+$ T cells in response to PBS or HDM. **a,** UMAP showing all fourteen lung CD4$^+$ T cell clusters identified. **b-d,** Defining the identity of the clusters in **a**. Dot plots showing the expression of selected markers (**b**), transcriptional regulators (**c**), and Th-related mediators and transcription factors (**d**). The size of each dot denotes the percentage of cells expressing the indicated gene for each cluster and the colour intensity of the dot represents the expression level of the gene.

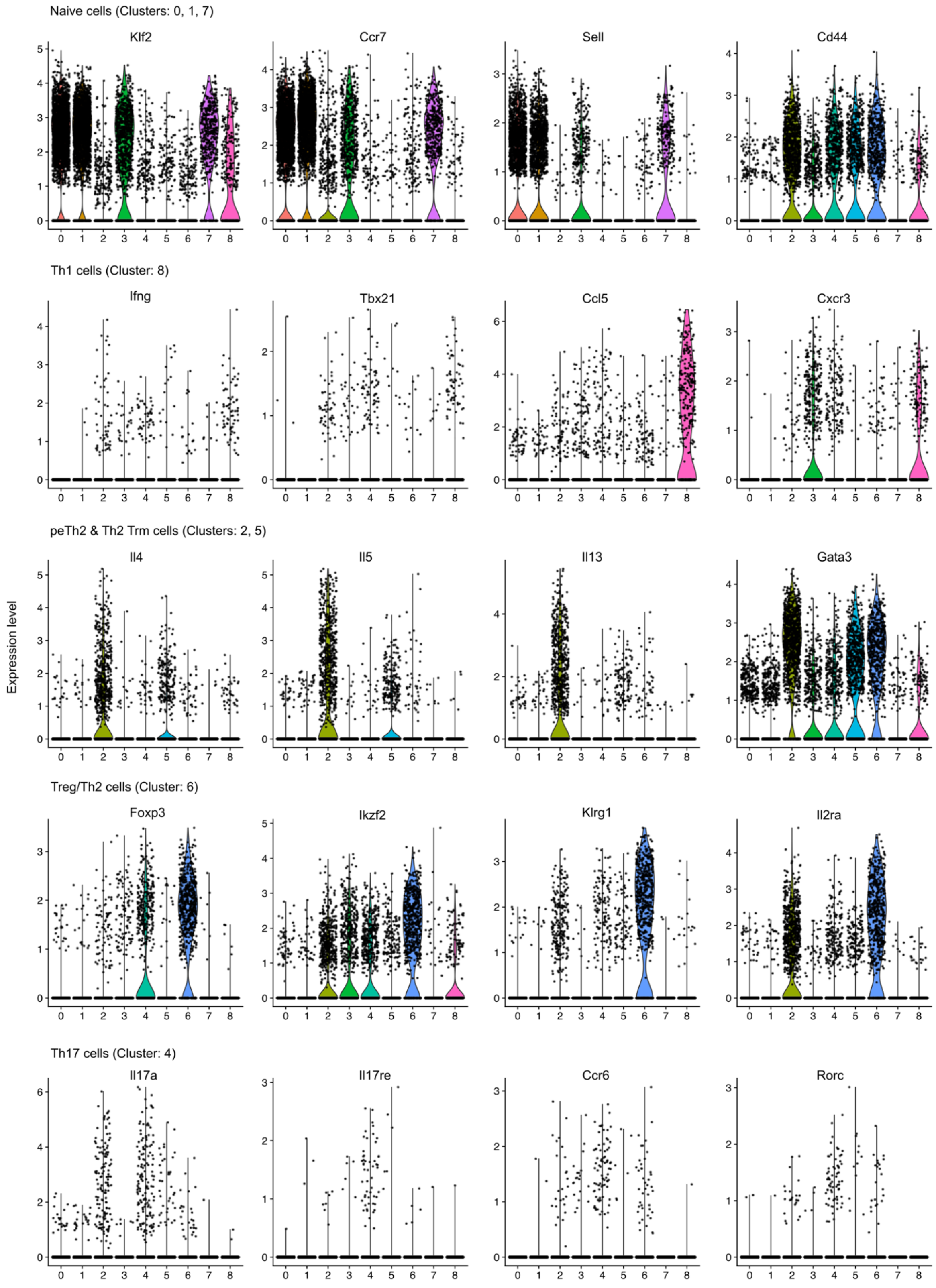

Supplementary Fig.3| Th lineage-specific markers and comparison of pTh2 cell subsets to published data obtained from airway pTh2 cell scRNA-seq. Violin plots of selected genes expressed by naïve CD4$^+$ T cells and Th-lineages.

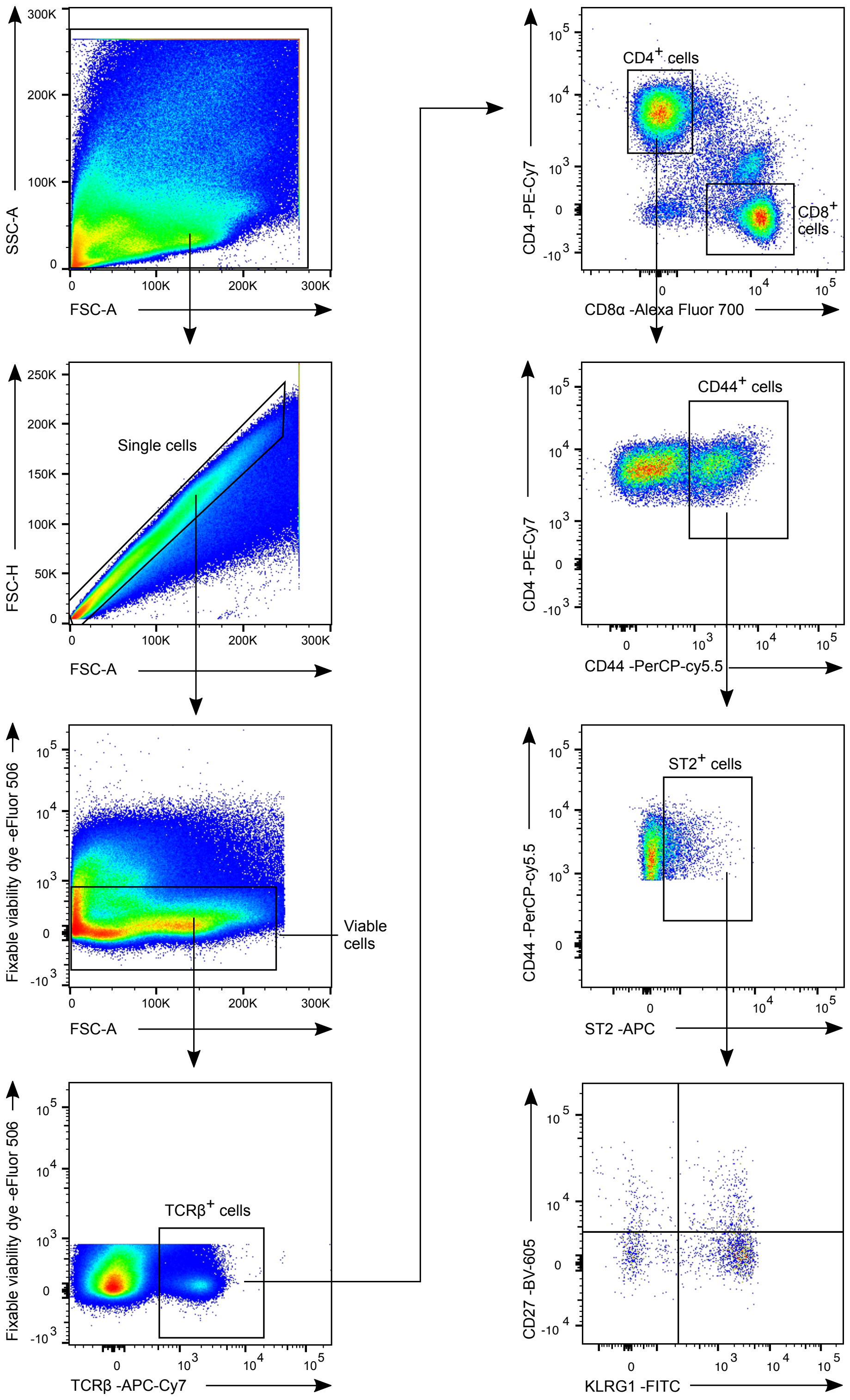

Supplementary Fig.4| Gating strategy for lung ST2^{+} Th cells. Representative flow cytometry plots showing the gating strategy for lung Th cells. The plots shown are from a WT mouse sensitised and challenged with HDM (as in Supplementary Fig. 1a). Single cells were obtained by gating away all the doublet cells. Next, we gated on the viable cells by excluding all dead cells from the single cells using a fixable viability dye. We then identified the T cells by gating on TCRβ^{+} cells. CD4^{+} T cells and cytotoxic CD8^{+} T cells in the TCRβ^{+} population were identified based on the expression of CD4 and CD8α, respectively. CD44 was used to exclude naïve CD4^{+} T cells and mark all Th (effector) cells within the CD4^{+} T cells. And all ST2^{+} cells were gated on the CD44^{+} cells. ST2^{+} cells were further gated based on CD27 and KLRG1 expression.

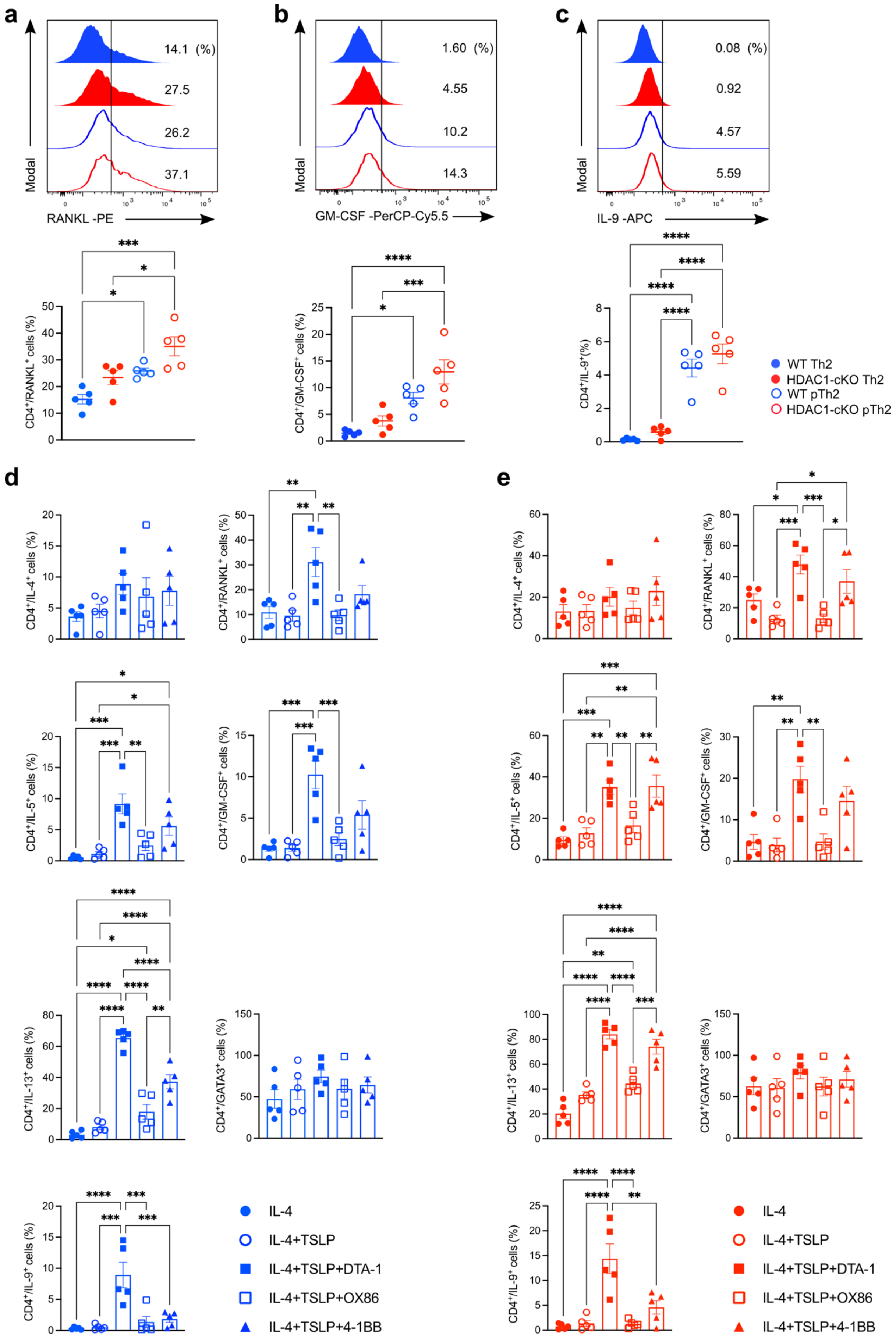
a
b
c
d
e
Modal
RANKL -PE
GM-CSF -PerCP-Cy5.5
IL-9 -APC
14.1 (%)
27.5
26.2
37.1
1.60 (%)
4.55
10.2
14.3
0.08 (%)
0.92
4.57
5.59
CD4+/RANKL+ cells (%)
CD4+/GM-CSF+ cells (%)
CD4+/IL-9+(%)
WT Th2
HDAC1-cKO Th2
WT pTh2
HDAC1-cKO pTh2
CD4+/IL-4+ cells (%)
CD4+/IL-5+ cells (%)
CD4+/IL-13+ cells (%)
CD4+/GATA3+ cells (%)
CD4+/IL-9+ cells (%)
IL-4
IL-4+TSLP
IL-4+TSLP+DTA-1
IL-4+TSLP+OX86
IL-4+TSLP+4-1BB

Supplementary Fig.5| Flow cytometric analysis of pathogenic mediators and comparison of TNFRSF members. a-c, Analyses of pathogenic mediators in *in vitro* generated pTh2 cells. Naïve $CD4^+$ T cells ($TCR\beta^+CD4^+CD62L^+CD44^-$) from WT and HDAC1-cKO mice were activated with anti-CD3 and anti-CD28 in the presence of Th2-promoting conditions (IL-4, IL-2, anti-IFN-γ, and anti-TGF-β) or pTh2-promoting conditions (IL-4, IL-2, TSLP, DTA-1, anti-IFN-γ, and anti-TGF-β) and cultured for 5 days. On day 5, we restimulated the cells with PMA and ionomycin in the presence of GolgiStop and GolgiPlug for 4 hours and performed cytokine analyses by flow cytometry. **a,** Histogram shows the expression of RANKL (top) and the frequency of Th cells expressing RANKL (bottom). **b,** Histogram shows the expression of GM-CSF (top) and the frequency of Th cells expressing GM-CSF (bottom). **c,** Histogram shows the expression of IL-9 (top) and the frequency of Th cells expressing IL-9 (bottom). **d,e,** Comparing the impact of TNFRSF members in inducing a pathogenic program in Th2 cells. We isolated naïve $CD4^+$ T cells ($TCR\beta^+CD4^+CD62L^+CD44^-$) from WT and HDAC1-cKO mice and activated them with anti-CD3 and anti-CD28 in the presence of Th2-promoting conditions (IL-4, IL-2, anti-IFN-γ, and anti-TGF-β; collectively termed IL-4), or IL-4+TSLP alone, or IL-4+TSLP+DTA-1, or IL-4+TSLP+OX86, or IL-4+TSLP+3H3. We cultured the cells for 5 days and restimulated them with PMA and ionomycin in the presence of GolgiStop and GolgiPlug for 4 hours before cytokine analyses by flow cytometry. **d,** Graphs showing the frequencies of pathogenic Th2 mediators and GATA3 in WT cells (blue). **e,** Graphs showing the frequencies of pathogenic Th2 mediators and GATA3 in HDAC1-cKO cells (red). Data are pooled from five independent experiments and presented as the mean ± SEM. Each symbol represents one mouse. Statistical analysis was performed using a one-way ANOVA with Tukey's multiple comparisons test. $^{*}P<0.05$, $^{**}P<0.01$, $^{***}P<0.001$, $^{****}P<0.0001$. TSLP, thymic stromal lymphopoietin; DTA-1, agonistic antibody against GITR (TNFRSF18); OX86, agonistic antibody against OX40 (TNFRSF4); 3H3, agonistic antibody against 4-1BB (TNFRSF9). Source data (**a-e**) are provided as a Source Data file.

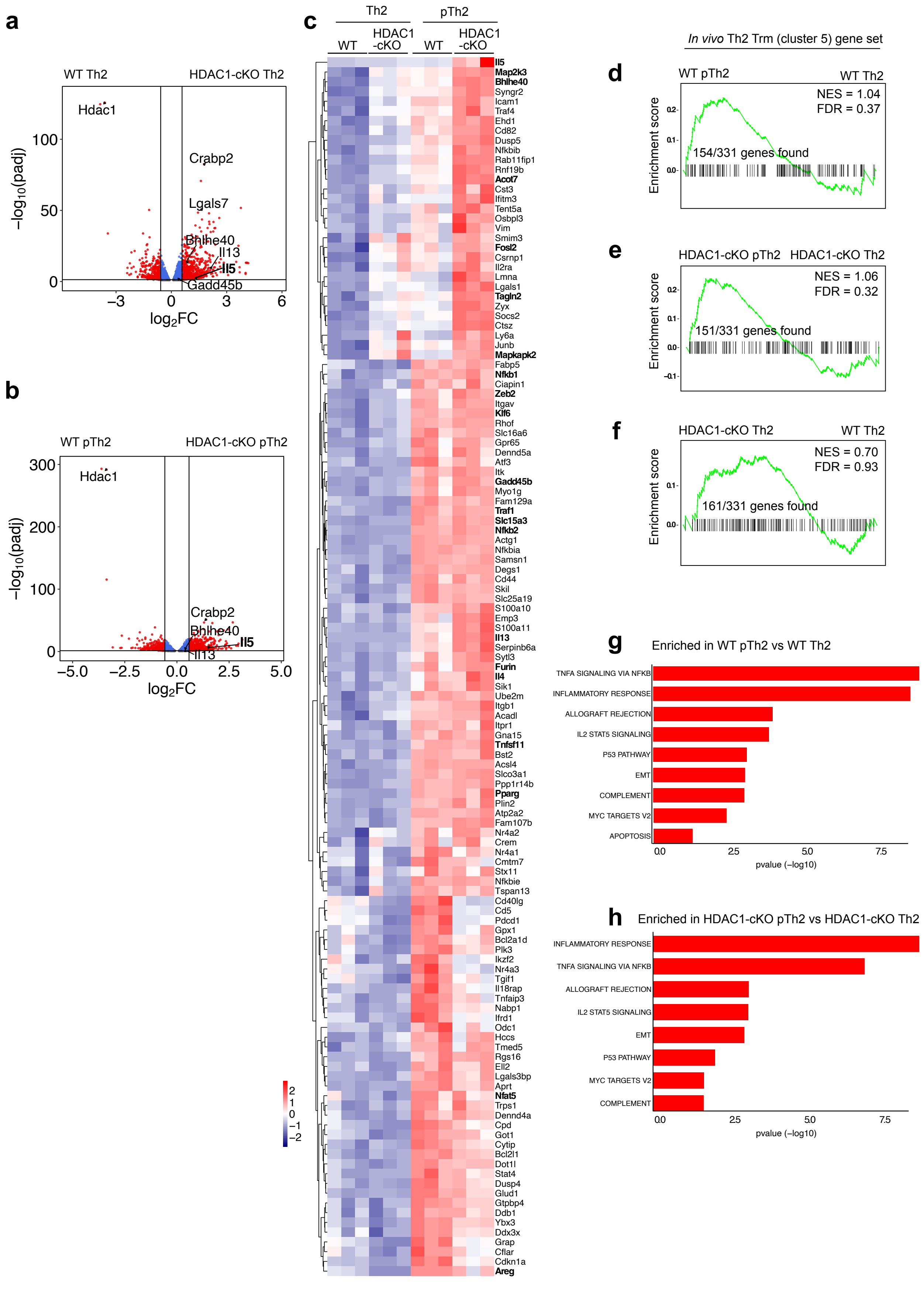

a
WT Th2
HDAC1-cKO Th2
Hdac1
Crabp2
Lgals7
Bhlhe40
Il13
Il5
Gadd45b
−log10(padj)
log2FC
b
WT pTh2
HDAC1-cKO pTh2
Hdac1
Crabp2
Bhlhe40
Il5
Il13
−log10(padj)
log2FC
c
Th2
pTh2
WT
HDAC1 -cKO
WT
HDAC1 -cKO
Il5
Map2k3
Bhlhe40
Syngr2
Icam1
Traf4
Ehd1
Cd82
Dusp5
Nfkbib
Rab11fip1
Rnf19b
Acot7
Cst3
Ifitm3
Tent5a
Osbpl3
Vim
Smim3
Fosl2
Csrnp1
Il2ra
Lmna
Lgals1
Tagln2
Zyx
Socs2
Ctsz
Ly6a
Junb
Mapkapk2
Fabp5
Nfkb1
Ciapin1
Zeb2
Itgav
Klf6
Rhof
Slc16a6
Gpr65
Dennd5a
Atf3
Itk
Gadd45b
Myo1g
Fam129a
Traf1
Slc15a3
Nfkb2
Actg1
Nfkbia
Samsn1
Degs1
Cd44
Skil
Slc25a19
S100a10
Emp3
S100a11
Il13
Serpinb6a
Sytl3
Furin
Il4
Sik1
Ube2m
Itgb1
Acadl
Itpr1
Gna15
Tnfsf11
Bst2
Acsl4
Slco3a1
Ppp1r14b
Pparg
Plin2
Atp2a2
Fam107b
Nr4a2
Crem
Nr4a1
Cmtm7
Stx11
Nfkbie
Tspan13
Cd40lg
Cd5
Pdcd1
Gpx1
Bcl2a1d
Plk3
Ikzf2
Nr4a3
Tgif1
Il18rap
Tnfaip3
Nabp1
Ifrd1
Odc1
Hccs
Tmed5
Rgs16
Ell2
Lgals3bp
Aprt
Nfat5
Trps1
Dennd4a
Cpd
Got1
Cytip
Bcl2l1
Dot1l
Stat4
Dusp4
Glud1
Gtpbp4
Ddb1
Ybx3
Ddx3x
Grap
Cflar
Cdkn1a
Areg
In vivo Th2 Trm (cluster 5) gene set
d
WT pTh2
WT Th2
NES = 1.04
FDR = 0.37
154/331 genes found
Enrichment score
e
HDAC1-cKO pTh2
HDAC1-cKO Th2
NES = 1.06
FDR = 0.32
151/331 genes found
Enrichment score
f
HDAC1-cKO Th2
WT Th2
NES = 0.70
FDR = 0.93
161/331 genes found
Enrichment score
g
Enriched in WT pTh2 vs WT Th2
TNFA SIGNALING VIA NFKB
INFLAMMATORY RESPONSE
ALLOGRAFT REJECTION
IL2 STAT5 SIGNALING
P53 PATHWAY
EMT
COMPLEMENT
MYC TARGETS V2
APOPTOSIS
pvalue (−log10)
h
Enriched in HDAC1-cKO pTh2 vs HDAC1-cKO Th2
INFLAMMATORY RESPONSE
TNFA SIGNALING VIA NFKB
ALLOGRAFT REJECTION
IL2 STAT5 SIGNALING
EMT
P53 PATHWAY
MYC TARGETS V2
COMPLEMENT
pvalue (−log10)

Supplementary Fig.6| Transcriptomic analysis of *in vitro* generated pTh2 cells. a-f, Transcriptome profiling of *in vitro* differentiated Th2 and pTh2 cells. Transcriptomic profiling from Th2 and pTh2 cells from WT and HDAC1-cKO mice. **a,b,** Volcano plots showing comparison between WT Th2 and HDAC1-cKO Th2 (**a**) and WT pTh2 and HDAC1-cKO pTh2 (**b**). The vertical and horizontal lines indicate Log2 Fold change of ≤ -0.58 and ≥ 0.58, and adjusted *P*-value < 0.05, respectively. For **a,b,** two-tailed *P*-values were obtained by DESeq2's Wald test and adjusted using the Bioconductor Independent Hypothesis Weighting package. **c,** Heatmap showing the expression profiles of WT pTh2 leading-edge genes (Fig. 7d) across *in vitro* differentiated Th2 and pTh2 cells from WT and HDAC1-cKO mice. The heatmap was generated using the DESeq2 normalised counts of the leading-edge genes (Supplementary Data 9). **d,** Enrichment plot showing a comparison of WT pTh2 cells and WT Th2 cells to lung Th2 Trm cells. **e,** Enrichment plot showing comparison of HDAC1-cKO pTh2 cells and HDAC1-cKO Th2 cells to lung Th2 Trm cells. **f,** Enrichment plot showing a comparison of HDAC1-cKO Th2 cells and WT Th2 cells to lung Th2 Trm cells. DEGs (adjusted *P*-value < 0.1; two-tailed *P*-values obtained by DESeq2's Wald test and adjusted using the Bioconductor Independent Hypothesis Weighting package) between WT pTh2 vs WT Th2, HDAC1-cKO pTh2 vs HDAC1-cKO Th2, and HDAC1-cKO Th2 vs WT Th2 (Supplementary Data 7) were used to compare with lung Th2 Trm gene set (DEGs; adjusted *P*-value < 0.05 based on Seurat's two-tailed Wilcoxon rank sum test with Bonferroni correction; Supplementary Data 2). **g,** GSEA of HALLMARK pathways upregulated (unadjusted *P*-value < 0.05) in *in vitro* generated WT pTh2 vs WT Th2. **h,** GSEA of HALLMARK pathways upregulated (unadjusted *P*-value < 0.05) in *in vitro* generated HDAC1-cKO pTh2 vs HDAC1-cKO Th2. For **d-h,** *P*-values were calculated using fgsea's adaptive multilevel splitting Monte Carlo scheme with Benjamini-Hochberg correction. Transcriptomic data are from three independent experiments. TNF, tumour necrosis factor; NFKB, nuclear factor kappa B; IL-2, Interleukin-2; STAT5, signal transducer and activator of transcription factor 5; EMT, epithelial-mesenchymal transition; FDR, false discovery rate; NES, normalised enrichment score.

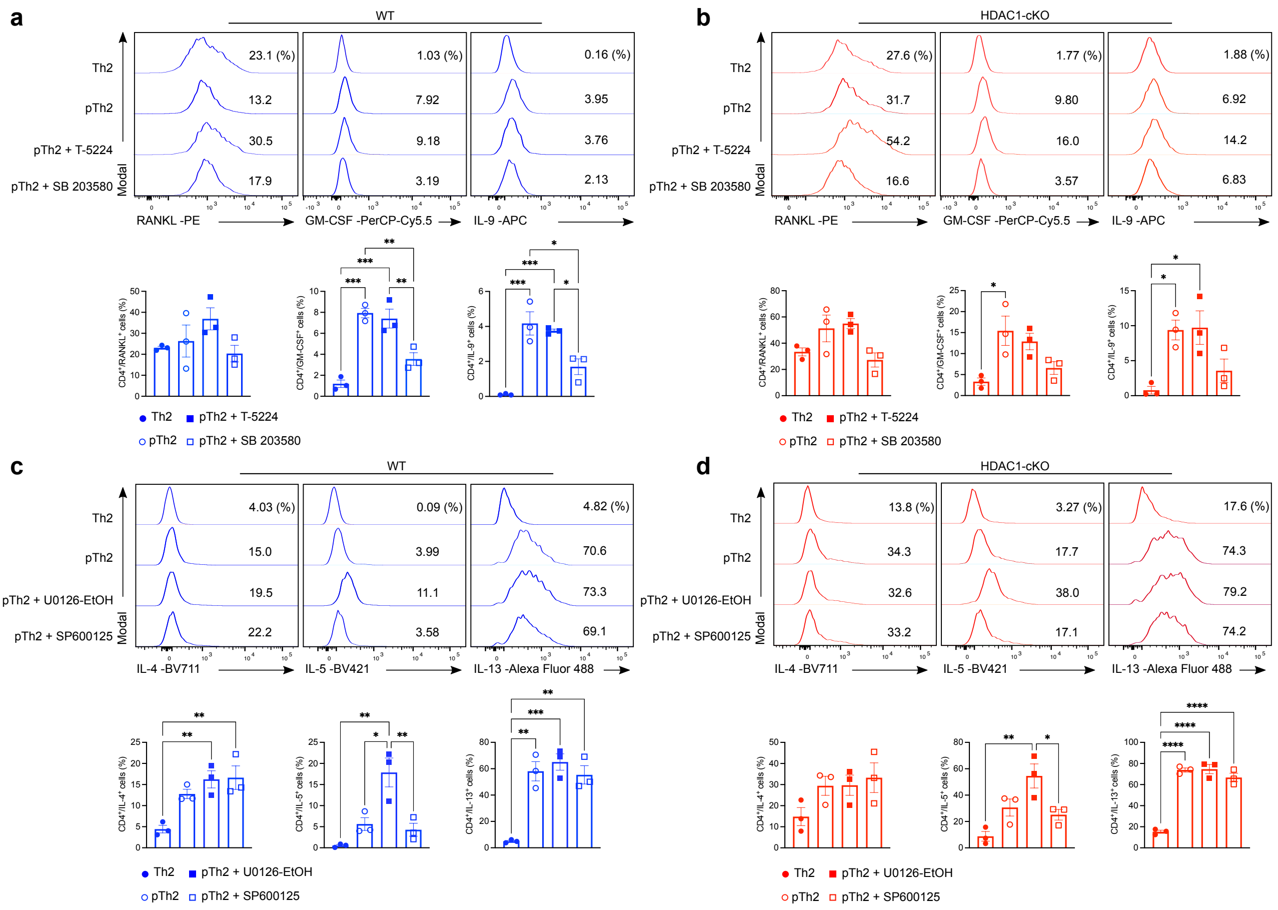

Supplementary Fig.7| Impact of inhibiting AP-1 and MAPKs on *in vitro* generated pTh2 cells. a-d, Naïve CD4$^+$ T cells from WT and HDAC1-cKO mice were differentiated under Th2-promoting conditions, or pTh2-promoting conditions alone, or pTh2 cells treated with an AP-1 inhibitor (T-5224; 10 μM), or pTh2 cells treated with a p38 MAPK inhibitor (SB 203580; 10 μM), and cultured for 5 days. On day 5, cells were restimulated with PMA and ionomycin in the presence of GolgiStop and GolgiPlug for 4 hours and cytokine analyses were performed by flow cytometry. **a,** Histograms show expression (top) and frequencies (bottom) of RANKL, GM-CSF, and IL-9, respectively, in WT cells (blue). **b,** Histograms show expression (top) and frequencies (bottom) of RANKL, GM-CSF, and IL-9, respectively, in HDAC1-cKO cells (red). **c,d,** Inhibition of ERK1/2 and JNK signalling in *in vitro* generated pTh2 cells. Naïve CD4$^+$ T cells from WT and HDAC1-cKO mice were differentiated under Th2-promoting conditions (IL-4, IL-2, anti-IFN-γ, and anti-TGF-β) alone, or pTh2-promoting conditions (IL-4, IL-2, TSLP, DTA-1, anti-IFN-γ, and anti-TGF-β) alone, or pTh2 cells treated with a MEK1/2 inhibitor (U0126-EtOH; 1 μM) which targets ERK1/2 signalling, or pTh2 cells treated with a JNK inhibitor (SP600125; 1 μM), and cultured for 5 days. On day 5, we restimulated the cells with PMA and ionomycin in the presence of GolgiStop and GolgiPlug for 4 hours and performed cytokine analyses by flow cytometry. **a,** Histograms show expression (top) and frequencies (bottom) of IL-4, IL-5, and IL-13, respectively, in WT cells (blue). **b,** Histograms show expression (top) and frequencies (bottom) of IL-4, IL-5, and IL-13, respectively, in HDAC1-cKO cells (red). Data are pooled from three independent experiments and presented as the mean ± SEM. Each symbol represents one mouse. Statistical analysis was performed using a one-way ANOVA with Tukey's multiple comparisons test. $^*P<0.05$, $^{**}P<0.01$, $^{***}P<0.001$, $^{****}P<0.0001$. TSLP, thymic stromal lymphopoietin; DTA-1, an agonistic antibody against GITR (TNFRSF18). AP-1, activator protein-1; MAPKs, mitogen-activated protein kinases; p38 MAPK, p38 mitogen-activated protein kinase; ERK, extracellular signal-regulated kinase; JNK, c-Jun N-terminal Kinase. Source data (**a-d**) are provided as a Source Data file.

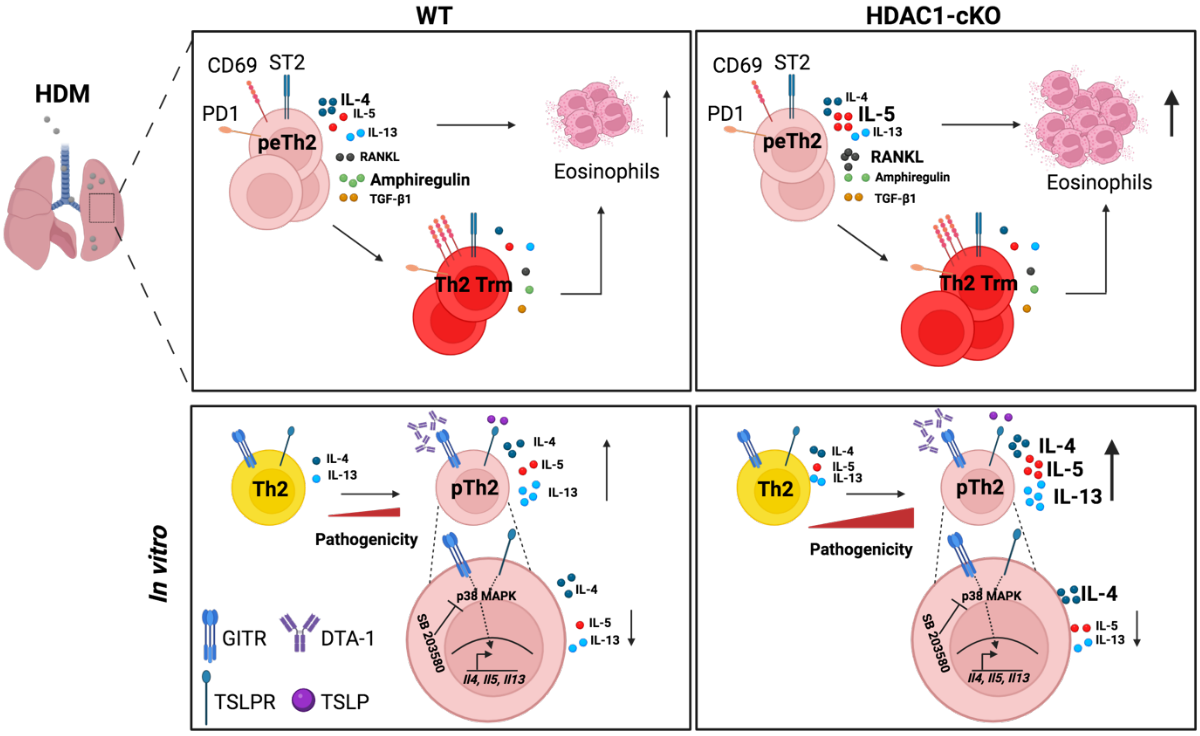

Supplementary Fig.8| Heterogeneity of pathogenic Th2 subsets and regulation of pathogenic Th2 differentiation by HDAC1. Scheme summarizing some of the main findings of the manuscript. Created in BioRender. Khan, M. (2025) https://BioRender.com/d56x498.
