# Supplementary material for: Single-cell and chromatin accessibility profiling reveals regulatory programs of pathogenic Th2 cells in allergic asthma
Source: Nat Commun. 2025 Mar 15;16:2565. doi: 10.1038/s41467-025-57590-3 (PMC11910648; doi:10.1038/s41467-025-57590-3)
Supplement: Supplementary file 2 — Description of Additional Supplementary Files [file 41467_2025_57590_MOESM2_ESM.pdf]

Description of Additional Supplementary Files

Supplementary Data 1: Total number of lung cells from each sample and their distribution across clusters obtained from scRNA-seq analysis**.**

Supplementary Data 2: Differentially expressed genes per cluster. *P*-values were calculated using Seurat's default two-tailed Wilcoxon rank sum test with Bonferroni correction.

Supplementary Data 3: Comparison of transcriptional signatures of peTh2 cells (cluster 2) and Th2 Trm cells (cluster 5) in this study to airway pathogenic Th2 cells (Day 15 Th2 cells from Tibbitt et al. (Immunity, 2019))**.** For all DEGs from Seurat, *P*-values were calculated using Seurat's default two-tailed Wilcoxon rank sum test with Bonferroni correction. For Gene set enrichment analysis (GSEA), *P*-values were calculated using fgsea's adaptive multilevel splitting Monte Carlo scheme with Benjamini-Hochberg correction.

Supplementary Data 4: Differentially expressed genes in peTh2 cells (cluster 2) vs all other clusters, and Th2 Trm cells (cluster 5) vs all other clusters. *P*-values were calculated using Seurat's default two-tailed Wilcoxon rank sum test with Bonferroni correction.

Supplementary Data 5: Differentially expressed genes in cluster 2 vs Cluster 5. *P*-values were calculated using Seurat's default two-tailed Wilcoxon rank sum test with Bonferroni correction.

Supplementary Data 6: GSEA for pathways dysregulated in peTh2 cells (cluster 2) vs all other clusters, and Th2 Trm cells (cluster 5) vs all other clusters. *P*-values were calculated using fgsea's adaptive multilevel splitting Monte Carlo scheme with Benjamini-Hochberg correction.

Supplementary Data 7: RNA-seq profiling of *in vitro* generated WT and HDAC1-cKO pTh2 and Th2 cells. Two-tailed *P*-values are based on DESeq2's Wald test and adjusted using the Bioconductor Independent Hypothesis Weighting package.

Supplementary Data 8: Comparison of transcriptional signatures of *in vitro* generated pTh2 cells to *in vivo* peTh2 cells (cluster 2) and Th2 Trm cells (cluster 5)**.** *P*-values were calculated using fgsea's adaptive multilevel splitting Monte Carlo scheme with Benjamini-Hochberg correction.

Supplementary Data 9: DEseq2 normalised counts (RNA-seq) of *in vitro* generated pTh2 and Th2 cells from WT and HDAC1-cKO cells.

Supplementary Data 10: Gene set enrichment analysis (GSEA) for pathways dysregulated in *in vitro* generated pTh2 cells. *P*-values were calculated using fgsea's adaptive multilevel splitting Monte Carlo scheme with Benjamini-Hochberg correction.

Supplementary Data 11: Proteomics profiling of *in vitro* generated WT and HDAC1-cKO pTh2 and Th2 cells. Two-tailed *P*-values were obtained by Limma moderated t-test with Benjamini-Hochberg correction.

Supplementary Data 12: List of antibodies used for flow cytometry analysis with clone, dilution, vendor, and fluorochrome.
