# Supplementary material for: Single-cell and chromatin accessibility profiling reveals regulatory programs of pathogenic Th2 cells in allergic asthma
Source: Nat Commun. 2025 Mar 15;16:2565. doi: 10.1038/s41467-025-57590-3 (PMC11910648; doi:10.1038/s41467-025-57590-3)
Supplement: Supplementary file 15 — Reporting Summary [file 41467_2025_57590_MOESM15_ESM.pdf]

nature portfolio

Corresponding author(s): Nicole Boucheron

Last updated by author(s): Feb 2, 2025

Reporting Summary

Nature Portfolio wishes to improve the reproducibility of the work that we publish. This form provides structure for consistency and transparency in reporting. For further information on Nature Portfolio policies, see our Editorial Policies and the Editorial Policy Checklist.

Statistics

For all statistical analyses, confirm that the following items are present in the figure legend, table legend, main text, or Methods section.

n/a	Confirmed	
☐	☒	The exact sample size (*n*) for each experimental group/condition, given as a discrete number and unit of measurement
☐	☒	A statement on whether measurements were taken from distinct samples or whether the same sample was measured repeatedly
☐	☒	The statistical test(s) used AND whether they are one- or two-sided<br>*Only common tests should be described solely by name; describe more complex techniques in the Methods section.*
☒	☐	A description of all covariates tested
☒	☐	A description of any assumptions or corrections, such as tests of normality and adjustment for multiple comparisons
☐	☒	A full description of the statistical parameters including central tendency (e.g. means) or other basic estimates (e.g. regression coefficient) AND variation (e.g. standard deviation) or associated estimates of uncertainty (e.g. confidence intervals)
☐	☒	For null hypothesis testing, the test statistic (e.g. *F*, *t*, *r*) with confidence intervals, effect sizes, degrees of freedom and *P* value noted<br>*Give P values as exact values whenever suitable.*
☒	☐	For Bayesian analysis, information on the choice of priors and Markov chain Monte Carlo settings
☒	☐	For hierarchical and complex designs, identification of the appropriate level for tests and full reporting of outcomes
☒	☐	Estimates of effect sizes (e.g. Cohen's *d*, Pearson's *r*), indicating how they were calculated

Our web collection on statistics for biologists contains articles on many of the points above.

Software and code

Policy information about availability of computer code

Data collection	-Flow cytometry samples were acquired on BD LSRFortessa (BD Biosciences). Cell sorting was carried out using BD FACSAria Fusion (BD Biosciences).<br>-For scRNA-seq, samples were sequenced on a NovaSeq 6000 instrument (Illumina) and processed using the Cell Ranger suite (6.1.2, 10X Genomics).<br>-RNA-seq samples were sequenced on a HiSeq 4000 instrument following a 50-base-pair, single-end recipe. Raw data acquisition (HiSeq Control Software, HCS, HD 3.4.0.38) and base calling (Real-Time Analysis Software, RTA, 2.7.7) were performed on-instrument, while the subsequent raw data processing off the instruments involved two custom programs based on Picard tools (2.19.2). In the first step, base calls were converted into lane-specific, multiplexed, unaligned BAM files suitable for long-term archival (IlluminaBasecallsToMultiplexSam, 2.19.2-CeMM). In a second step, archive BAM files were demultiplexed into sample-specific, unaligned BAM files (IlluminaSamDemux, 2.19.2-CeMM).<br>-Quantitative mass spectrometry analysis was performed on an Orbitrap Eclipse mass-spectrometer (Thermo Fisher) coupled to an UltiMate 3000 Dual LC nano-HPLC System (Dionex, Thermo Fisher Scientific).<br>-ATAC-seq samples were sequenced on a NovaSeq 6000 instrument (Illumina, San Diego, CA, USA) in a 100-base pair paired-end configuration. Chromatin accessibility mapping by ATAC-seq was done in three biological replicates. NGS reads in unaligned BAM files were converted into FASTQ format with samtools145, NGS adapter sequences were removed via fastp146 (0.23.2, GTCTCGTGGGCTCGG) and the reads were aligned to the GRCm38 (UCSC Genome Browser mm10) assembly with Bowtie2147 (2.4.4, --very-sensitive, --no-discordant, --maxins 2000), before deduplicating with samblaster148 (0.1.24). BED-files were generated from the BAM-files using bedtools bamtobed, and further into a file compatible with scATAC-seq analysis software (atac_fragments.tsv.gz) using a custom Java program (https://github.com/henriksson-lab/bulkatac2fragments). Signac 1.13 was used for dimensional reduction, visualisation and differential accessibility testing149. Peak calling was performed via built-in MACS2150. Motifs were called using the JASPAR2020 database, and motif activity was computed using chromVAR (https://www.nature.com/articles/nmeth.4401). The motif activity correlation (difference) were calculated using Rfast::correls.

Data analysis	Flow cytometry:<br>FlowJo (v.10.8.1, Tree Star)<br><br>Analysis of non-omics data:<br>GraphPad Prism (v.9.3.1)<br><br><br>scRNA-Seq:<br>R (v.4.1.2),<br>Seurat package (v.4.0.6)<br>DoubletFinder (v.2.0.3)<br>ggplot2 (v.3.4.0)<br>EnhancedVolcano (v.1.12.0)<br>scCustomized<br>DeepVenn [http://www.deepvenn.com/]<br>The Molecular Signatures Database (MSigDB) (v.7.5.1)<br><br>RNA-seq analysis:<br>-"Spliced Transcripts Alignment to a Reference" (STAR, 2.7.5a)<br>-Bioconductor (3.11)<br>-GenomicAlignments (1.24.0) package<br>-DESeq2 (1.28.1) package<br>-ashr (2.2.-47) package<br>-Independent Hypothesis Weighting (IHW, 1.16.0) package<br>-ggplot2 (v.3.4.0)<br>-EnhancedVolcano (v.1.12.0)<br>-pheatmap (v.1.0.12)<br>-fgsea package (v.1.20.0)<br>-MSigDB (v.7.5.1)<br><br>Proteomics:<br>-FreeStyle 1.7 software (Thermo Scientific)<br>-MaxQuant software (version 1.6.17.0)<br>-Uniprot database (release 2021.03; with isoforms)<br>-R (4.1.0)<br>-LIMMA (3.50)<br>-ggplot2 (v.3.4.0)<br>-EnhancedVolcano (v.1.12.0)<br>-pheatmap (v.1.0.12)<br><br>ATAC-seq analysis:<br>-samtools-0.1.x package<br>-fastp 0.23.2 package<br>-Bowtie v1.2.3<br>-Samblaster v. 0.1.24<br>-Signac 1.13<br>-MACS (2.2.9.1)<br>-chromVAR<br>-Rfast::Correl

For manuscripts utilizing custom algorithms or software that are central to the research but not yet described in published literature, software must be made available to editors and reviewers. We strongly encourage code deposition in a community repository (e.g. GitHub). See the Nature Portfolio guidelines for submitting code & software for further information.

Data

Policy information about availability of data

All manuscripts must include a data availability statement. This statement should provide the following information, where applicable:

- Accession codes, unique identifiers, or web links for publicly available datasets
- A description of any restrictions on data availability
- For clinical datasets or third party data, please ensure that the statement adheres to our policy

Raw single-cell sequencing data were submitted to ArrayExpress (#E-MTAB-13089) and RNA-seq and ATAC-seq data to Gene Expression Omnibus with the accession codes GSE235803 and GSE280390 respectively. The mass spectrometry proteomics data have been deposited to the ProteomeXchange Consortium via the PRIDE partner repository with the dataset identifier PXD042975. The authors declare that the data supporting the findings of this research are available in the article, the Supplementary Material, or on request from the corresponding author. All information will be available to editors and reviewers during reviewing/revision period and to the public after acceptance of the manuscript.

Research involving human participants, their data, or biological material

Policy information about studies with human participants or human data. See also policy information about sex, gender (identity/presentation), and sexual orientation and race, ethnicity and racism.

Reporting on sex and gender	No human samples or subjects were used in this study
Reporting on race, ethnicity, or other socially relevant groupings	No human samples or subjects were used in this study
Population characteristics	No human samples or subjects were used in this study
Recruitment	No human samples or subjects were used in this study
Ethics oversight	No human samples or subjects were used in this study

Note that full information on the approval of the study protocol must also be provided in the manuscript.

Field-specific reporting

Please select the one below that is the best fit for your research. If you are not sure, read the appropriate sections before making your selection.

[x] Life sciences [ ] Behavioural & social sciences [ ] Ecological, evolutionary & environmental sciences

For a reference copy of the document with all sections, see nature.com/documents/nr-reporting-summary-flat.pdf

Life sciences study design

All studies must disclose on these points even when the disclosure is negative.

Sample size	Samples for scRNA-seq experiments were obtained by pooling 3 mice per group.<br>RNA-seq experiments consisted 3 biological replicates per group.<br>Proteomics experiments consisted 4 biological replicates per group.<br>ATAC-seq experiments consisted of 3 biological replicates per group.
Data exclusions	In the proteomics analysis, one WT pTh2 sample was excluded from the analysis due to poor sample quality.
Replication	All experiments were conducted using biological replicates, and data were compiled from independent experiments.
Randomization	No randomization was performed. Mice were assigned based on genotypes.
Blinding	The authors were not blinded.

Reporting for specific materials, systems and methods

We require information from authors about some types of materials, experimental systems and methods used in many studies. Here, indicate whether each material, system or method listed is relevant to your study. If you are not sure if a list item applies to your research, read the appropriate section before selecting a response.

Materials & experimental systems

n/a	Involved in the study	
[ ]	[x]	Antibodies
[x]	[ ]	Eukaryotic cell lines
[x]	[ ]	Palaeontology and archaeology
[ ]	[x]	Animals and other organisms
[x]	[ ]	Clinical data
[x]	[ ]	Dual use research of concern
[x]	[ ]	Plants

Methods

n/a	Involved in the study	
[x]	[ ]	ChIP-seq
[ ]	[x]	Flow cytometry
[x]	[ ]	MRI-based neuroimaging

Antibodies

Antibodies used	Antibody (Clone, Vendor, Dilution, Colour)<br>TCRb (H57-597, eBioscience, 1:100, APC-Cy7)

	ST2 (RMST2-2, eBioscience ,1:100, APC)<br>ST2 (RMST2-2, eBioscience, 1:100, Percp-e710)<br>FoxP3 (FJK-16s, eBioscience, 1:200, APC)<br>IL-13 (eBio 13A, eBioscience, 1:200, Alexa Fluor 488)<br>Ly6G (1A8, BioLegend, 1:200, BV421)<br>CD19 (6D5, BioLegend, 1:500, BV605)<br>CD11b (M1/70, BioLegend, 1:200, BV786)<br>MHC class II (AF6-120.1, BioLegend, 1:100, FITC)<br>Siglec F (S17007L, BioLegend, 1:200, PE)<br>F4/80 (BM8, BioLegend, 1:100, PE-Cy7)<br>CD11c (N418, BioLegend, 1:400, APC)<br>CD4 (RM4-5, BioLegend, 1:400, PE-Cy7)<br>CD44 (IM7, BioLegend, 1:200, PerCp-Cy5.5)<br>KLRG1 (2F1/KLRG1, BioLegend, 1:200, FITC)<br>CD27 (LG.3A10, BioLegend, 1:200, BV605)<br>GATA3 (16E10A23, BioLegend, 1:200, BV421)<br>CD69 (H1.2F3, BioLegend, 1:200, BV421)<br>PD1 (29F.1A12, BioLegend, 1:200, BV786)<br>PD1 (29F.1A12, BioLegend, 1:200, BV711)<br>IL-4 (11B11, BioLegend, 1:200, BV711)<br>IL-5 (TRFK5, BioLegend, 1:200, BV421)<br>IL-9 (RM9A4, BioLegend, 1:200, APC)<br>RANKL (IK22/5, BioLegend, 1:200, PE)<br>GM-CSF (MP1-22E9, BioLegend, 1:200, PerCp-Cy5.5)<br>CD62L (MEL-14, BD Biosciences, 1:200, FITC)<br>CD25 (PC61, BD Biosciences, 1:200, APC)<br>CD8a (53-6.7, BD Biosciences, 1:200 Alexa, Fluor 700)<br>GATA3 (L50-823, BD Biosciences, 1:200, PETexas-Red)<br>CD44 (IM7, BD Biosciences, 1:200, BV786)
Validation	Validation was provided by the manufacturer.

Animals and other research organisms

Policy information about studies involving animals; ARRIVE guidelines recommended for reporting animal research, and Sex and Gender in Research

Laboratory animals	Hdac1 flox/flox (HDAC1f/f)<br>CD4-Cre<br>IL-13tdTomato
Wild animals	No wild animals were used.
Reporting on sex	Both male and female mice were used
Field-collected samples	No field samples were used.
Ethics oversight	Animal husbandry and experiments were reviewed and approved by the Institutional Review Board of the Medical University of Vienna and approved by the Austrian Ministry of Economy and Science (BMWFW-2020-0.547.902) and performed as per the guidelines of the Federation of European Laboratory Animal Science Associations.

Note that full information on the approval of the study protocol must also be provided in the manuscript.

Flow Cytometry

Plots

Confirm that:

- [x] The axis labels state the marker and fluorochrome used (e.g. CD4-FITC).
- [x] The axis scales are clearly visible. Include numbers along axes only for bottom left plot of group (a 'group' is an analysis of identical markers).
- [x] All plots are contour plots with outliers or pseudocolor plots.
- [x] A numerical value for number of cells or percentage (with statistics) is provided.

Methodology

Sample preparation	Sample preparation is provided in methods.

Instrument	BD LSRFortessa<br>FACSAria Fusion
Software	FACS Diva software and FlowJo
Cell population abundance	N/A
Gating strategy	For in vivo experiments, gating strategy is provided in Supplementary Fig.4.<br>In in vitro experiments, cells were selected using forward and side scatter followed by doublet exclusion. Viable cells were then identified using a fixable viability dye (eFluor 506, eBioscience), followed by gating on viable CD4+ cells for further analysis.

[x] Tick this box to confirm that a figure exemplifying the gating strategy is provided in the Supplementary Information.
